# 

**Published:** 1872-05

**Authors:** 


### Books and Pamphlets Received.

History of Medicine from the earliest ages to the commencement of the nineteenth Century. By Robley Dunglison, M. D., L.L. D., etc. Arranged and edited by Richard J. Dunglison, M. D. Philadelphia: Lindsay and Blakiston, 1872. Buffalo: Theodore Butler & Son (by subscription only.)

A practical treatise on the diseases of women. By T. Gaillard Thomas, M.D. etc. Third edition, enlarged and thoroughly revised, two hundred and forty-six illustrations on wood. Philadelphia: Henry C. Lea, 1872. Buffalo: Theodore Butler & Son.

Clinical Lectures on the Diseases of Women. By Sir James T. Simpson, Bart.,

M. D., D. C. L., etc. Edited by Alexander R. Simpson, M. D. etc. New York: D. Appleton & Co. 1872 Buffalo: Breed, Lent & Co.

A Treatise on disease of the Bones. By Thomas M. Markoe, M. D., etc. New York: D. Appleton & Co. 1872. Buffalo: Breed, Lent & Co.

Forty-sixth Annual Report of the Surgeons of the Massachusetts charitable Eye anu Ear Infirmary, Feb.. 1872. Boston: James Campbell, 1872.

Quarterly Summary of the Transactions of the College of Physicians of Philadelphia. From May 18, 1870 to Feb. 7, 1872 inclusive.

Irrigations of Ice Water as a means of arresting Hæmorrhage in cases of Placenta Prævia. By W. A. Baldwin, M. D., Montgomery, Ala. Reprint from Richmond and Louisville Medical Journal, April 1872.

Remarks on Stricture Urethra of extreme calibre, with cases and a description of new instruments for their treatment. By F. N. Otis, M. D., etc. Reprint from New York Medical Journal, Feb., 1872.

On the Physiology of Syphilitic Infection. By F. N. Otis, M. D., etc. Reprint from "The Medical Gazette."

Notes on Fluid Extract of Senega, Rhubarb, commercial Bicarbonate of Soda, Chloral, etc. By Edward R. Squibb, M. D., of Brooklyn. Reprinted from the Proceedings of the American Pharmaceutical Association for 1871.

An Investigation concerning the Mechanism of the Ossicles of Hearing, and the Membrance of the Round Window. By Charles Burnett, M. D., etc. Reprinted from the Archives of Ophthalmology and Otology. Vol. II. No. 2 1872.

The Ethics of the Medical Profession. Read before the Sacremento Society for Medical Improvement. By Joseph T. Montgomery, M. D.

Statistics and Information relative to the Trade and Commerce of Buffalo for the year 1871. With a list of Officers, Members, etc., of Board of Trade. By Wm. Thurstone, Secretary.

Earth as a Topical Application in Surgery. By Addinell Hewson, M. D., one of the attending Surgeons to the Pennsylvania Hospital. With four Photo-Relief Illustrations, octavo pp. 309. Philadelphia: Lindsay & Blakiston, 1872. Buffalo: Breed, Lent & Co.

The Urine and its Derangements. With the application of Physiological chemistry to the Diagnosis and Treatment of constitutional as well as local diseases. By George Harley, M. D., F. R. S. with illustrations, octvao pp. 334. Philadelphia: Lindsay & Blakiston, 1872. Buffalo: Breed Lent & Co.

Dr. Rigby's Obstetric Memoranda. Fourth edition Revised and enlarged. By Alfred Meadows, M. D. Philadelphia: Lindsay & Blakiston, 1872. Buffalo: Breed, Lent & Co.

Memoranda on Poisons. By the late Thomas Hawkes Tanner, M. D., F. L. S. Philadelphia: Lindsay & Blakiston 1872. Buffalo: Breed, Lent & Co.

Second Annual Report of the Board of Directors of the Manhattan Eye and Ear Hospital. New York.

Proceedings of the Homœopathic Medical Society of Ohio. Seventh Annual session at Cincinnati Ohio, 1871.

Half-hour Recreations in Popular Science. Dana Estes, Editor. No. 3. Spectrum Analysis Explained. Illustrating its uses to science, and including the Theory of sound, heat, light and color. By Profs. Schellen, Roscoe and Huggins. Boston: Lee & Shepard, 1872. Buffalo. Herger & Ulbrich.

Der Kindergarten in Amerika. New York: E. Steiger, 1872.

Clinical experiences in private practice. Therapentical effects of Opium illustrated. By Z. C. McElroy, M. D., Zanesville, Ohio. Reprint from Medical Archives, May, 1872.

Tables I, II, III, IV, V, VI, VII, VIII, IX, U. S. Censuses 1870, 1860, 1850 showing morality of the United States by states and territories, with distinction of sex, percentage of deaths to population, etc.

A Treatise on the diseases of Infancy and Childhood. Second edition enlarged and thoroughly revised. By J. Lewis Smith, M. D., etc. Philadelphia: Henry C. Lea, 1872. Buffalo: T. Butler & Son.

THE BUFFALO

Medical and Surgical Journal

PUBLISHED MONTHLY,

Containing Original Articles, Reports of Medical Societies and Hospitals, Editorial, Reviews, Correspondence, Army News, etc., etc.; including the usual variety of Medical Periodical Publications. Specimen copies sent on application.

Address Buffalo Medical & Surgical Journal, Buffalo, N. Y.

TERMS OF SUBSCRIPTION, - - $3.00 PER YEAR, IN ADVANCE.

